# Challenge, fear and pride: nursing students working as nurses in COVID-19 care units

**DOI:** 10.1080/17482631.2022.2100611

**Published:** 2022-07-21

**Authors:** Cristina Gómez-Moreno, Eva García-Carpintero Blas, Pablo Lázaro, Esperanza Vélez-Vélez, Gregorio Jesús Alcalá-Albert

**Affiliations:** a Fundación Jiménez Díaz School of Nursing of Madrid, Autonomous University of Madrid, Madrid, Spain; b Health Research Department Independent Health Services Researcher, Madrid, Spain; c Nursing Department, Alfonso X El Sabio University, School of Medicine, Madrid, Spain

**Keywords:** COVID-19, nursing students, learning, qualitative research, challenge, transition role, phenomenological design

## Abstract

**Context and Purpose:**

During the crisis caused by the COVID-19 pandemic in Spain, students in the year of undergraduate degree were hired to provide care assistance support in hospitals. The purpose of the study is to explore their experiences of their premature professional incorporation into patient care in a pandemic situation.

**Methods:**

A descriptive phenomenology research study was conducted. Data were collected in two phases: 1) Two focus groups and 2) Ten in-depth individual semi-structured interviews between July and August 2021.

**Results:**

Twenty-two Nursing students from a Madrid University School of Nursing participated. All worked in COVID hospitalization units, 6 in intensive care units. Four main categories were identified. *Student-professional nurse transition, Learning*, *Hospital integration* and *Emotions.*

**Conclusion:**

Despite all the fears and negative emotions, the nursing students do not regret the decision to accept a contract to work as a healthcare professional in the COVID-19 pandemic. They feel that the pandemic has allowed them to see life from another perspective and with other priorities, strengthening their vocation to nursing.

## Introduction

In December 2019, cases of atypical pneumonia caused by severe acute respiratory syndrome due to coronavirus 2 (SARS-CoV-2) began to be diagnosed in Wuhan, a city in the Chinese province of Hubei (Zhang et al., 2020). Progressively, cases of coronavirus disease 2019 (COVID-19) began to appear in many countries. On 11 March 2020, more than 118,000 cases of COVID-19 had been declared in 114 countries, with 4,291 deceased patients, for which the World Health Organization (WHO) declared a pandemic situation (General Director World Health Organization, 2020). By the end of January 2021, almost 100 million cases and more than two million deaths were registered worldwide (Worldometers, 2021).

In Europe, the first non-imported case of COVID-19 was declared in Germany on 28 January 2020. In Spain, the first case appeared on January 31. Since then, the virus has spread rapidly, affecting the country severely. The Spanish Government declared on 14 March 2020, the State of Alarm throughout the national territory to curb the health emergency caused by SARS-COV 2.

Spanish hospitals began to receive patients who occupied more and more hospital and Intensive Care Units (ICUs) beds. Given the hospital care overload and the shortage of healthcare personnel, the Spanish Ministry of Health requested the voluntary incorporation of the last academic year nursing students to help the National Health System respond to the COVID-19 crisis under a contract known as “Contract of relief for health professionals” (Centro Nacional de Epidemiología. Ministerio de Sanidad. Gobierno de España, 2020).

Historically, nursing professionals have been at the forefront of the fight against epidemics and pandemics (Johnson, 2021). The COVID-19 pandemic has shown the health system’s high relevance, especially for nursing professionals, at the front care line. Nursing is the workforce with the most significant number of health professionals participating at all levels of care in controlling this pandemic (Cáceres-Rivera, 2021). In the UK, retired nurses and university nursing students have been an active part of this workforce (Galvin et al., 2020). Several authors have reported that health professionals have experienced post-traumatic stress and other undesirable consequences from working in pandemics (Chandler-Jeanville et al., 2021; Hayter & Jackson, 2020; Li et al., 2021). Specifically, some studies show that nurses who worked with COVID patients experienced emotional burdens and mental disturbances (Karimi et al., 2020).

Published research on the COVID-19 pandemic and its effects on college students is emerging rapidly. Some studies have analysed their knowledge and attitudes to face outbreak situations, mainly in Asian countries (Elrggal et al., 2018), but few have been carried out in Europe (Monforte-Royo & Fuster, 2020). In England, there were difficulties in establishing the roles of students and delimiting their competencies, and a lack of experience in the management of pandemics and similar emergencies was observed (Hayter & Jackson, 2020; Swift et al., 2020). Swift et al. (2020) and Li et al (2021), suggest that students felt pressure to support their peers and society when entering the clinical practice environment. Other authors questioned whether our students were prepared to face such a challenge that, on the one hand, meant moving to the professional role and, on the other, addressing the pandemic they experienced (Cervera-Gasch et al., 2020). In addition, they reflect that the COVID-19 outbreak has affected students both in their personal and professional lives (Casafont et al., 2021).

Many students recruited as healthcare aids to support nurses in Spain had completed only part of their clinical training without rotating through units later considered first-line. Furthermore, they continued their studies simultaneously (Monforte-Royo & Fuster, 2020). All this has been able to directly influence their learning and personal life and how they have experienced the change of role from student to professional nurse (Galehdar et al., 2020; García-Martín et al., 2021). Although some authors already addressed incorporating these last-year students into clinical practice during the pandemic (Casafont et al., 2021; Collado-Boira et al., 2020; Monforte-Royo & Fuster, 2020; Velarde-García et al., 2021), students experiences need further analysis. Also is important and aspects to be improved on nursing education during a pandemic (Backes et al., 2020; Haslam, 2021; Pereira et al., 2020).

## Objectives

The objectives of this study were to explore: 1) the experiences of last-year nursing students hired as healthcare assistance in response to the COVID-19 crisis; 2) how such experience impacted their learning process; 3) how they lived their role change, and 4) their feelings during this process.

## Materials and methods

A descriptive qualitative study framed in phenomenology explored what nursing students experienced during the COVID-19 outbreak. Qualitative research takes into account the natural contexts in which individuals or groups function to provide insights into real-world problems (Moser & Korstjens, 2018). Specifically, phenomenology is a methodological, theoretical orientation, in which it is about “seeing” the phenomena from those who experience them. Its objective is to discover the essence of the phenomenon (Barbera & Inciarte, 2012). According to Mortari (2019), phenomenology applied to empirical research requires researchers to explore facts of the perspective of the participants. His narratives reveal the empirical qualities of a experience that cannot be separated from the context of the phenomenon.

The study setting was a tertiary-level teaching hospital in Madrid, Spain.

The participants were fourth-year undergraduate nursing students, 2019/2020 academic year, from Schools of Nursing and Faculty of Nursing of different universities of Madrid, hired as healthcare aids before finishing their clinical training to respond to the COVID pandemic. The students started working under a “contract of relief/support for health professionals”.

The inclusion criteria were fourth-year undergraduate nursing students hired with a relief contract during the outbreak who worked as healthcare aids during the Pandemic with Covid-19 patients for at least one month. They agreed to participate in the study after signing the informed consent voluntarily.

Were not accepted (exclusion criteria), students with previous work experience in the healthcare field, those who rejected the “contract for health professionals”, students who, during the health crisis, worked with COVID patients outside the hospital setting (primary care setting, residences, out-of-hospital centres, schools), who were hired as healthcare aid personnel but not in COVID units and finally students with an academic degree in a health science other than nursing (medicine, psychology, physical therapy).

Data were collected in two phases: 1) Two focus groups and 2) Ten in-depth individual semi-structured interviews between July and August 2021.

In the first phase, convenience sampling was carried out. The selection of participants was based on their ability to provide relevant information to respond to the study objective (Creswell & Poth, 2016; Moser & Korstjens, 2018), choosing the students from the Fundación Jiménez Diaz School of Nursing—Autonomous University of Madrid (FJD-UAM)

In the second phase, the sample was expanded with snowball sampling techniques to locate participants from other hospitals in the Community of Madrid through the participants included in the first phase. Information redundancy was achieved, reaching data saturation in participant 22 (Moser & Korstjens, 2018). There were no dropouts.

### Data collection

In the first phase, two discussion groups were conducted. In the second phase, 10 in-depth individual semi-structured interviews were carried out between July and August 2021. A prior sociodemographic questionnaire was completed by the participants (Table 1). The codes were constructed using the following characters: G for the discussion groups and E for the interviews, followed by a correlative number from 1 to the total number of participants.

**Table 1.** Participants’ sociodemographic profile.

Discoussion groups	GENDER	AGE	UNIT	PREVIOUS ROTATION FOR THE UNIT
G1P1	F	22	HOSPITALIZATION	NO
G1P2	M	22	HOSPITALIZATION	NO
G1P3	M	22	HOSPITALIZATION	NO
G1P4	M	23	ICU	YES
G1P5	F	22	HOSPITALIZATION	NO
G1P6	F	22	ICU	YES
G1P7	F	22	HOSPITALIZATION	YES
G2P1	F	22	HOSPITALIZATION	YES
G2P2	F	23	HOSPITALIZATION	Yes (paediatrics)
G2P3	F	23	HOSPITALIZATION	NO
G2P4	F	22	ICU	YES
G2P5	F	22	HOSPITALIZATION	NO
INTERVIEWS	GENDER	AGE	UNIT	PREVIOUS ROTATION FOR THE UNIT
E1	F	24	REA-ICU	REA NO ICU YES
E2	F	22	HOSPITALIZATION	NO IN MOST OF THEM
E3	M	23	HOSPITALIZATION	NO
E4	F	23	HOSPITALIZATION	NO
E5	F	22	ICU	YES
E6	F	22	HOSPITALIZATION	NO
E7	F	22	HOSPITALIZATION	NO
E8	M	22	HOSPITALIZATION	NO
E9	F	22	ICU, REA, SURGERY ROOM, PAEDIATRICS	YES, IN SOME OF THEM
E10	M	24	HOSPITALIZATION	NO

Discussion groups are a type of conversational technique that studies the intersubjectivity of the group; that is, how the participants interact and especially the common conclusions about opinions, ideas, or feelings (Rapley, 2011). A discussion guide aligned with the study’s objectives and framed in the existing literature was used at the group meeting. The discussion guide was structured in an introduction followed by the development of the questions that led to the key themes (Table 2). Both groups finished by summarizing the points discussed, and a time was left for those participants wanting to provide extra information not collected or without time to comment on. During the meeting, if a question generated debate or controversy or the speaking turns were not respected, one of the researchers was the moderator. The other researcher, the observer, collected field notes during the groups’ debate. The first discussion group had seven participants, and the second, five.

**Table 2.** Thematic areas of the discussion groups.

Themes	Questions
Experience	How has your experience been during the COVID-19 pandemic?
Integration in the team	How did you feel when you started in the hospital? How was your integration in the team or the unit?
Student-professional role	What was your role during the contract? How did you feel?
Learning	Did you feel that you had received all the necessary training to face the care of patients with COVID? How has your learning been during it?

In a second phase, in-depth semi-structured interviews were conducted with open questions using the information previously obtained in the discussion groups (Table 3), thus expanding the information on the addressed topics (Moser & Korstjens, 2018).

**Table 3.** Thematic areas of the interviews and open questions.

Themes	Questions
Experience	How has your experience been during the COVID-19 pandemic? How did you feel when you got home? How has facing the pandemic changed you personally or at work?
Team	How has your experience been during the COVID-19 pandemic? What feelings do you remember from the first day of work? How did you feel upon arrival at the unit? Did you feel integrated within the team? During the day to day in the hospital, what feelings were the most predominant?
Student-professional role	When she accepted the contract as a health worker, was she informed of her duties and responsibilities? What was your role in the pandemic? During clinical practice, were you under the supervision of a professional tutor or, on the contrary, were you alone in the care of the patients?
Learning	Do you think you have entered the labour world with less training than promotions in other years? Or, on the contrary, do you think that working during the pandemic has made you gain experience and skill? Did you receive any previous training?

The Covid-19 pandemic has accelerated the need to explore alternative data collection methods for qualitative research in nursing. Due to the outbreak, the discussion groups and interviews were conducted through the Microsoft Teams video conferencing platform (Hernan-García, 2021). The discussion groups lasted approximately 90 min, and the interviews took between 60–70 min. Both the discussion groups and the interviews were transcribed verbatim. Data collection was done through video recordings with audio and field notes from the researcher. These field notes were obtained during all the interviews and discussion groups, collecting information on the non-verbal language of the participants, recording essential information and incidents during its development (Creswell & Poth, 2016). Confidentiality was ensured by consecutively numbering each interview and focus group and subsequently removing identifying information from the transcripts.

### Data analysis

Five researchers with experience in health science research participated in the data analysis, and an external researcher allowed data triangulation. The analysis was carried out following Giorgi’s proposal, according to which five stages are followed in data processing (Giorgi, 1997):

1. Information gathering through semi-structured interviews.
2. Reading carefully after verbatim transcription of the interviews.
3. Decomposition, to identify the meaning units and relevant categories according to the study objectives and the information obtained.
4. Organization and enumeration through a coding process
5. Interpretation, systematization, and summary of the data, to disseminate the results.

In the transcription phase, the audios and annotations of the interviews and the discussion groups were literally transcribed for their subsequent interpretation. In this part of the analysis, alphanumeric codes were used to anonymize the participants (Table 1). Subsequently, the data reduction was carried out, carefully reading the texts to select the most relevant information according to the study’s objectives. The software Atlas it was used as computer support to analyse both, the interviews and the discussion groups (Soratto et al., 2020).

To guarantee the study quality, the Consolidated Criteria for Reporting Qualitative Research (COREQ) (Tong et al., 2007) were followed. In addition, criteria of rigour and scientific quality of the qualitative studies proposed by Lincoln et al. (2011) were used. These criteria were: Credibility, controlled by applying triangulation of the researchers during the analysis, as well as triangulation of the data collection methods with the use of discussion groups, interviews, and field notes by the researchers; Transferability, through a detailed description of the method used and the phenomenon under study; Dependency and confirmability, guaranteed by checking the data collected in the primary documents transcribed with the participants; and finally, in addition, an external evaluator was in charge of evaluating the study, focusing on aspects related to the applied design and study methods.

## Ethical considerations

This study respects the fundamental principles of the *Declaration of Helsinki of the Council of Europe on Human Rights and the Biomedicine*. Also, the *UNESCO Universal Declaration on the Human Genome* and the *Convention for the Protection of Human Rights and Dignity of the Human Being with regard to the Application of Biology and Medicine.*

The project, identified with the code PIC114-21_FJD, was approved by Research Ethics Committee of Fundación Jiménez Díaz Hospital in compliance with the bioethical principles of autonomy, justice, beneficence and non-maleficence.

Before the meetings, the participants were given an information sheet about the data collection tools and purpose of the study and the informed consent, which they had to sign in advance, giving their agreement for data collection by recording the discussion groups and interviews, and registering the notes taken by the researchers.

All information has been handled anonymously, complying with the Organic Law on Data Protection and Guarantee of Digital Rights 3/2018. The data collected has only been used for content transcription and interpretation in the context of the present study.

## Results

The 22 participants were between 21 and 24 years old, 16 were women, and 16 came from the Fundación Jiménez Díaz School of Nursing—UAM (FJD-UAM). All worked in COVID hospitalization units, 6 in ICUs (Table 1). The duration of the contracts was between one and three months.

**Figure 1.**
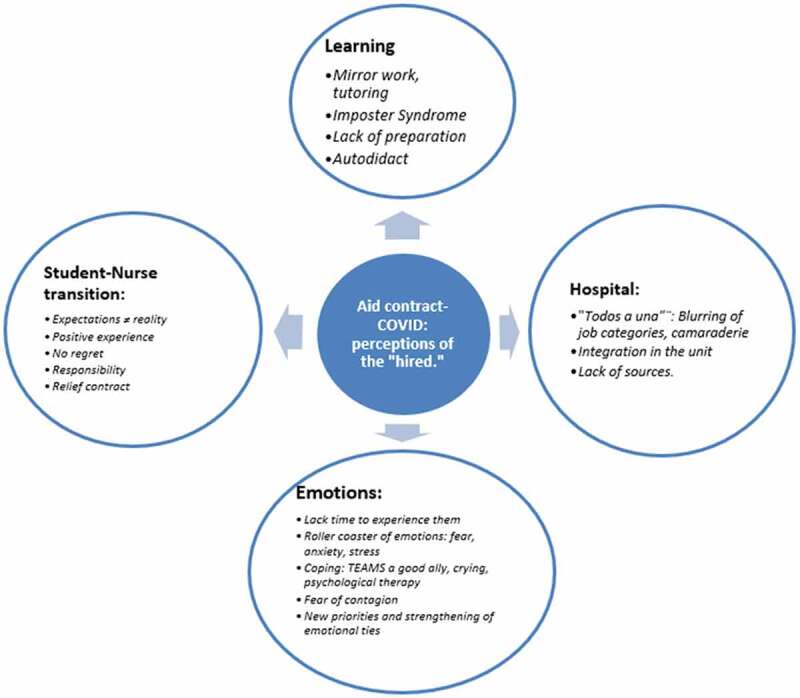
Challenges faced. Most frequent categories and codes.

After the data analysis, four main categories were identified concerning students’ perceptions: Student-nurse transition, learning process, hospital integration, and emotions. The topics were divided into ten subcategories (Table 4); the most frequent codes have also been extracted from each category as challenges faced (Figure 1).

**Table 4.** Categories and subcategories matrix.

CATEGORIES	SUBCATEGORIES
Transition student-nurse	Functions
	Decision
	Responsibility
Learning	Autonomous learning
	Feeling unprepared
	Tutoring
Hospital	Integration in the unit
	Equipment
Emotions	Feelings
	Family
	Coping strategies
	New priorities
	Psychic sequelae

### Student—professional nurse transition: expectations from the aid contract vs. reality

The transition from student to professional can be measured as a multidimensional approach. It results from living one of the most critical changes in a person’s life. Clinical training for the nursing profession is essential for job placement, especially when they have not completed clinical practices and job placement is amid a pandemic.

The students who accepted the relief contract had many doubts about their competencies and role in the hospital since there were no precedents for this situation. Some participants reported that the conditions initially communicated to them were not met; for example, initially, they were told they would not contact COVID patients but clean units.

They also told us we were not going to be in contact with COVID patients that we were always going to be out … and that was not fulfilled at all. G2P4.

At all times, the students felt that they wanted to help, but they were not clear if they would perform as professionals or as students. On many occasions, this role was not uniform or firm, depending on the units where they were located.

No, that is, they told you where you were to go, period. Supposedly you were going to be a support to the nurse in charge. However, the reality is that later you have patients assigned to your care. G2P1.

It was not my case. I know of student colleagues who worked without supervision, but I did not. The truth is that, in my case, the contract was respected at all times because I did what is, literally, a support function. Well, probably just once I worked unsupervised. E4.

As for whether they were faced with the moral obligation to accept the job, all participants said they agreed to face the pandemic because they felt the need to help, feeling that they have contributed to improving the current situation of the pandemic like the rest of the health professionals.

Sure … it was a grain of sand, so to speak, I mean … well … we contribute the same as any other person who was in the COVID can contribute, but, as a whole, the group was the one that did something, you know? That is, not just us. G1P4.

I believe that, in general, the contribution has been from all of us. No matter how much you put good faith and good intention into this, I think one by oneself can contribute little. I mean, it has been something great, and I think it’s been so because it has been a general contribution from all over the world. So … I don’t care who has been on the front line or doing paperwork or making calls; each person in their own way. E4.

In addition, none of the participants regrets the decision made to accept the healthcare assistant contract.

No, I don’t regret it at all, at all. In other words, for me, it was almost all positive. From a terrible situation, the personal experience was very positive. I will never regret it. I will always remember it, my first work experience. E7.

### Learning: “mirror work” vs. “impostor syndrome”

Regarding whether the participants received prior training when facing the pandemic, all reported not having received any preparatory courses; only some received a training video from their university on how to appropriately dress and undress the Personal Protective Equipment gear. In general, the entire process was carried out autonomously and self-taught, based on many occasions on trial and error, or by watching videos recommended by other professionals, or by the professionals themselves on site.

They couldn’t give us training when even they didn’t know what was going on. I think they were improvising on the fly, and we were part of that improvisation. G2P3.

This situation also accelerated the students learning process by increasing their knowledge, although they felt unprepared for everything they faced.

But you have no choice, you learn a lot and, well, as a result of … then and such that we are again now with COVID because now I am learning a lot … too. So, I think I have learned more than lost. E1.

In my case, I did feel a bit like impostor syndrome, that I was not there … and that I did not have enough knowledge to be there caring for patients. G2P3.

Even so, the students think it was a unique and unrepeatable learning opportunity professionally, gaining confidence and maturing. They feel that after this situation, they can cope with everything.

It is true that after facing this situation we lived in, we see ourselves with more strength to face new things. G1P1.

Regarding whether they had a professional tutor to supervise activities, there were differences among participants depending on the hospital in which they were working.

At least in my case we were alone, alone in the face of danger. G1P2.

You were under the supervision of the tutor, but we indeed worked in a mirror way. EIP.3

### Hospital: “disappearance of job categories” “lack of material”

All participants from discussion groups and individual interviews agree that they were received with open arms when they arrived at the destination unit and, generally, felt recognized and integrated within the team.

I didn’t have any problems, and whatever I needed, they talked about it and, well, they tried to help you as much as possible, so I did feel integrated, I mean, I felt part of the team, you were not “the trainee kiddo”. E10.

Concerning the above, many said that one of the things they had noticed is that the pandemic had managed to bring people closer together, support each other, improve camaraderie, and everyone started working as a team and not just as a group.

There were no longer ranks, I mean, we were all at that, together, it did not matter if you were a guard, nurse, assistant or doctor, it did not matter, we were exactly the same. We were all there to do the same thing; the companionship was great. Everybody was supporting you, and you were supporting them, constant support, you know?; because in the end, you knew it was the only thing you had. E9.

Lack of material was a global issue. Face masks had to be reused for weeks, reducing their effectiveness time to a few hours. In addition, the few masks they received were not as effective as they should be, leaving healthcare professionals and the participants of this study unprotected.

I was with an FFP2 … Well, I will remember this my whole life … An FFP2 for a whole month, possibly a bit more than a month; they were sterilized! Which I think was useless. From the sterilization, they came out wrinkled, with fallen rubber bands, holding it with staples … Well, that was shameful! It was truly shameful … EIP7.

The pandemic was also a challenge for the participants from the nurse-patient point of view since they were on the front line and had to see and cope with tough times that they had never faced or at least not so closely, such as patients’ death.

People who were the age of your father or your grandfather, choking. And you could not do anything, nothing al all; because they could not resist the prone, they could not endure, they did not tolerate it and … I have seen people who have died on top of the food … very beastly, very beastly … (nodding). But, hey, it is what there is and there were; that is to say, there is a type of patient that you knew was going to die. E10.

During the day-to-day in the hospital, one of the most repeated feelings was the frustration of seeing that patients took a long time to improve, increasing their hospital stay, making the nurse-patient relationship more important than ever.

A lot of frustration, for all the stuff you carried on your back, what your colleagues carried on their back, the deaths, the ill patients … a huge emotional load. On top of that, the ICU was … as I told you before, we made calls and video calls with our own mobiles so that patients could see their families! E9.

### Emotions: a roller coaster of feelings, coping, family relationship, therapies

On a personal level, the students experienced many types of emotions, predominantly fear, uncertainty, and physical and psychological exhaustion.

The night before, I couldn’t sleep at all. I lay on the bed thinking that I was going to start working the next day and, above all, in a place where they had told me that it was hell, to be careful, everyone was afraid of it. G1P6.

All participants reported feeling nervous before starting work, stressed, and anguish while working.

Above all that, no one has said anything, well, maybe it’s just me, but after the shift, I always kept thinking, or perhaps I’m still thinking, what could I have done wrong, if I had missed something … It was very stressful because you didn’t get to everything and wanted to cover many things. You have heard so many times during your studies must support your patient, listen to him … . But you can’t; you don’t have time. If you listen to a patient, then you have another to do, and in the end, you are incomplete; at least, I felt a little incomplete. G1P6.

The feelings and emotions of the participants did not end in the hospital when they finished their shift. Arriving home was a critical moment for them, as were the relationships with their families and friends. Most of the participants reported feeling the fear and concern of their relatives for them.

Yes, very scared … in fact, eh … my grandparents called me every day. My parents asked me every day about the situation at the hospital. The truth is that my whole family, not just my parents, were worried about me. E2.

In addition, some participants highlighted the feeling of loneliness since family and friends could not fully understand what they were experiencing.

I decided to stay in a hotel. I stayed there for a month in total, and the truth is that it was like … it was very sad because I felt very lonely (laughing). At work was where I felt best. I usually arrived an hour earlier, and everyone asked me what I was doing. I felt alone the rest of the day. I needed to socialize. G2P5.

Participants felt, at times, they could not tell their families the whole truth about what they were seeing and experiencing for fear of their reaction.

I did not spend time with them, nor did I eat or be with them. I felt lonely and bad. Today I have had sixteen deaths, and I cannot tell my family because it will worry them; besides, I cannot tell them that of the fifty there (he makes the gesture in quotation marks), sixteen were under my care.!. EIP6.

Some students expressed being unaware of everything they were experiencing while working due to the high care load. All the feelings came to them, and they were able to assimilate everything they had been through when they could stop a bit and even take some time off.

I think that when I stopped, uh, well in August, I asked five days off. Luckily, they gave them to me, and I think it was to stop and … well, just the moment of doing nothing (laughs), yes, I noticed a bit of anxiety. I mean, I think it is later when you realize things or your overwhelm and such … your anxiety. E1.

The fear of being an asymptomatic carrier was another feeling reported by the students, making some feel dirty. The concern of infecting their relatives made some of them decide to change their residence for at least a time, going to hotels or second homes.

I asked for a hotel, and I stayed for a month. To tell you the truth, it was like … it was very sad, I felt very lonely, but hey, the day I returned home I remember it as one of the happiest of my life. G2P5.

I felt dirty, so when I got home, I undressed and went to the shower because I felt like I had all the virus inside my nails, in my hair … horrible. As soon as I took a shower, I took my clothes, put them in the washing machine … pff, that was chaos in the mornings. E2.

Stress and overwhelm were the most repeated feelings among the participants. Nevertheless, each one tried to handle it in the best way they could. Among the tools most used to manage stress were talking with family and friends, in some cases through video conferences, or venting through tears. In the most severe cases, Psychological therapy or even tranquilizers prescription was needed.

I am not a person who keeps everything into myself so that I would talk to my mother. I would tell her about it. I would talk to my colleagues; I felt very understood. My partner is also a healthcare worker and also very understanding. E9.

Yes, I started going to the psychologist in September because of everything … I just couldn’t do it anymore because of everything. G1P2.

The lived experience made participants value and improve relationships, mainly family relationships. This whole situation made them value the moments spent together and to open up more emotionally with family and friends.

Now I make plans with my family on weekends, that is, before I was just friends and studying, now I’m going to have an aperitif with my parents on Saturdays, why? Because I value and love their company and their closeness much more. uh … it’s true, friends come and go and judge you and you do … and the family doesn’t. G2P2.

## Discussion

The first wave of the COVID-19 pandemic caused a shortage of qualified nurses in Spain. The main objective of this research was to study the experiences of final year nursing students hired as health care in response to the COVID-19 crisis. Our findings have important repercussions at the health and educational level, being relevant for universities and health institutions. In addition, this study also shows the coping strategies of students in this situation.

The findings show the lack of consensus regarding their role in the team and specific functions during the pandemic. Previous studies (Casafont et al., 2021; Velarde-García et al., 2021) already showed that the role and tasks were not defined and were adapted as needs arose.

The transition from student to professional is experienced as a stressful event. This tough process requires adapting to a new role and requires many skills at a multidimensional level. As Velarde-García et al. (2021) indicates, the first year of work is challenging for recent graduates, who may feel insecure about their skills and knowledge. Some studies show that a stipulated number of patients should be assigned based on recent graduates’ skills and abilities to make this transition easier. They also should receive comprehensive guidance and support from their colleagues (Pimmer et al., 2019; Suarez-Garcia et al., 2018). The present study results show that the participants experienced a transition from student to nurse rashly since they acquired that responsibility almost forcibly due to the emergency and magnitude of the crisis confronted.

They also reported feeling continuous stress, anxiety, fear, and uncertainty due to facing something unknown. This fear added fear of infecting their relatives since most of the participants lived with their families during the pandemic and felt anxious about taking the virus home. Previous studies have already recognized these fears (Algunmeeyn et al., 2020; Collado-Boira et al., 2020; Tan et al., 2020; Velarde-García et al., 2021).

Regarding their learning process, it is observed that students have been learning by trial and error, on many occasions without adequate tutoring and doing it autonomously and self-taught. These findings are similar to other studies (Velarde-García et al., 2021). However, Rodríguez-Monforte et al. (2021) reflects that students worked during the pandemic under tutoring and supervision until they acquired sufficient security. Despite adversity, our students consider that the situation they have experienced has provided them with a unique learning opportunity. They also feel more confident, prepared, and self-confident after working as a first-aid worker during the pandemic. In addition, they value the experience; thanks to it, they learned to face adversity and increased their personal and professional growth.

A recent study describes that last-year-nursing students who had to face the pandemic could feel morally pressured to a large extent to accept an aid contract due to the messages received through social networks (Gómez-Ibáñez et al., 2020). Our results show that the opinion of their family and environment was more important for the students. They did not question being helpful during the crisis, choosing to study nursing, and feeling useful. Gómez-Ibáñez et al. (2020) describes that students who have found themselves in this situation show more significant commitment to society and have consolidated their vocation as a professional. However, Rodríguez-Monforte et al. (2021) reflects that this very abrupt and premature transition caused by the pandemic could influence the students’ decision to remain in the nursing profession.

Like other authors (Taylor et al., 2017; Velarde-García et al., 2021) we share the responsibility of universities and health care institutions regarding the learning and care of our students. We must help them in their learning by supporting their professional development, especially in demanding situations such as the pandemic experienced.

Similar to other studies (Barrett, 2022; Casafont et al., 2021; Palacios-Ceña et al., 2022) these results can help nursing schools train their students to future health crises, implementing simulation laboratories and clinical situations that integrate theory and practice. All this would help to improve the abilities of the students and their emotional strategies. On the other hand, hospitals must strengthen their emotional support strategies that they provide to their healthcare professionals. From our results, we believe there should be institutional support. The mentorship by veteran nurses is crucial to facilitate the management of conflicts and dilemmas and avoid learning in solitude and at a forced pace. It is also important to teach student nurses to apply self-care strategies by including students in specific training programmes.

A study’s limitation is that our findings may not be extrapolated to nursing students from other settings or to students from other health professions who have worked during the COVID 19 pandemic, both due to the scope of the study and the type of design. Likewise, the interviews and discussion groups were conducted using an online digital platform given the health situation, which may have modified the amount, type, and subtleties of personal interaction between participants and researchers and could have influenced the results obtained.

A strength of the study is that robust qualitative methodology techniques, such as discussion groups and interviews, have allowed the results to be triangulated and reflect varied and even different perspectives and experiences.

Despite the limitations, results can contribute to understanding nursing students’ experiences and learning difficulties and identify their attitudes and experiences regarding the role they have played during the Pandemic of COVID-19.

## Conclusion

During the Covid-19 crisis, the nursing students hired as healthcare aids to support nurses experienced a rush in their learning, and their role change was lived with anxiety and stress. They felt that they did not have enough knowledge to face this situation. They had to carry out self-taught and autonomous learning on many occasions without a tutoring process that gave them security.

Fear of contagion, self and other, was a prevalent feeling. The pandemic strengthened family bonds, and most participants found the most significant support in their families.

Despite all the fears and negative emotions, the nursing students do not regret the decision made (to accept a contract to work as a healthcare professional in the COVID-19 pandemic) and feel that the pandemic has allowed them to see life from another perspective and with other priorities, strengthening their vocation to nursing.

Nursing students have been vital resources for our health system and our society when needed, and this applies to many countries. Regardless of the lack of preparedness perceived by the student to respond to a pandemic, they show a high level of altruism, and they had shown to be a valuable resource that needs to be tapped. Now it is time for us, both academics and health authorities, to reciprocate their efforts. It is necessary to implement the necessary improvements in training and security measures, not only because it affects the health and safety of the patient but also because they will be fundamental pieces in future pandemics.

This study is an implicit claim for further training in intensive care, emergencies, and disasters. It is also an implicit statement of the need to incorporate high-fidelity clinical simulation teaching methodologies, which offer excellent results.
